# Difficulty distinguishing benign notochordal cell tumor from chordoma further suggests a link between them

**DOI:** 10.1186/1470-7330-14-4

**Published:** 2014-04-22

**Authors:** Jennifer Kreshak, Frédérique Larousserie, Piero Picci, Stefano Boriani, Joseph Mirra, Biagio Merlino, Eugenio Brunocilla, Daniel Vanel

**Affiliations:** 1 Istituto Ortopedico Rizzoli, Via del Barbiano 1/10, 40136 Bologna, Italy; 2 Hopital Cochin, Paris, France; 3 Dipartimento di Scienze Radiologiche, Università Cattolica del Sacro Cuore, Policlinico "A. Gemelli", Largo Agostino Gemelli 8, 00168 Roma, Italy; 4 Dipartimento di Medicina Specialistica, Diagnostica e Sperimentale, U.O. di UROLOGIA, Via P.Palagi n. 9, 40100 Bologna, Italy

**Keywords:** Benign notochordal cell tumor, Chordoma, Computed tomography, Magnetic resonance imaging

## Abstract

**Background:**

Much discussion about benign notochordal cell tissue in vertebrae has centered on the nature of its relationship, if any, to chordoma. Often referred to as benign notochordal cell tumors (BNCTs), these lesions have unique morphological features, however, differentiating between notochordal cells in discs, BNCT, and chordoma can be difficult. They are described as radiologically distinct from chordoma, with lysis, contrast enhancement, and a soft tissue mass indicating chordoma.

**Methods:**

All chordomas diagnosed at our institution, the Istituto Ortopedico Rizzoli (Bologna, Italy), prior to 2008 were reviewed, yielding 174 cases. Five were limited to bone; one was a recurrent chordoma without original data available. The remaining four were re-evaluated in detail.

**Results:**

There were three women and one man, aged 33–57 years (mean, 48 years). Two were BNCTs and two were mixed lesions containing BNCT and chordoma. On computed tomography, all were radiopaque with areas of lysis. One BNCT was heterogeneous on magnetic resonance imaging, enhancing after contrast. Microscopically, one BNCT had a well-defined cystic area with a sclerotic border. The other had a minute atypical area; it recurred as chordoma. The mixed lesions had areas of definitive BNCT, definitive chordoma, and atypical areas that did not meet the criteria for either. The atypical areas in all three cases ‘blended’ with areas of chordoma or BNCT.

**Conclusion:**

These cases illustrate the ongoing challenges in differentiating between BNCT and chordoma. All had unique imaging features; three had atypical microscopic areas blending with BNCT or chordoma, strengthening the argument for a relationship between the two entities and supporting the idea that some BNCTs may progress to chordoma. Our study dispels the notion that any single radiologic criterion used to distinguish between chordoma and BNCT is reliable, opening the discussion as to whether or how to monitor BNCTs.

## Background

Intraosseous benign-appearing notochordal tissue has been observed in isolated fetal and adult spines during the last century [1-3], but only in the last three decades has it been well-characterized and explored in greater detail. Contemporary work called on previous studies by pathologists, anatomists, and embryologists, raising myriad questions about the natural history of the notochord and the origin of all notochordal lesions. Important case reports and series questioned whether this intraosseous notochordal tissue represented true notochordal rests or de novo lesions, whether it was benign or malignant [4-10]. At each turn, terminology changed, from ecchordosis physalifora vertebralis, to giant notochordal rest, to the now widely-used benign notochordal cell tumor (BNCT) [4,6,8,9]. Central to the discussion was the nature of the relationship, if any, between this benign-appearing tissue and chordoma. Several authors reported cases indicating an association between BNCT and chordoma [11-16] as others highlighted clear radiologic and histopathologic criteria for distinguishing between them [6-10,16,17].

Our objective was to identify BNCTs previously diagnosed as chordoma in order to better characterize their radiologic and pathologic characteristics and to better delineate any possible relationship between them. What we found demonstrates that the differences between BNCT and chordoma are not as simple as was once thought.

## Methods

The work was approved by the Institutional review board of the Rizzoli Institute. We searched the database at our institution for all cases of chordoma diagnosed prior to 2008; after 2008 BNCT was a well-known entity to us and was considered as a separate diagnosis when appropriate. One hundred seventy-four chordomas were found. Of these, 169 had a soft-tissue mass. Five were limited to bone, one of which was a recurrent chordoma sent from an outside institution; detailed information regarding the original lesion was not available. The remaining four cases were re-evaluated in detail, including review of the charts, imaging, and pathology. As all cases were originally diagnosed as chordoma, each underwent vertebrectomy and the entire vertebra had been available for study. Many patients at this institution come by referral, retaining their original films and all but one of these patients were diagnosed before digital imaging was adopted; as a result, not all imaging performed was presently available.

## Results

**Figure 1:**
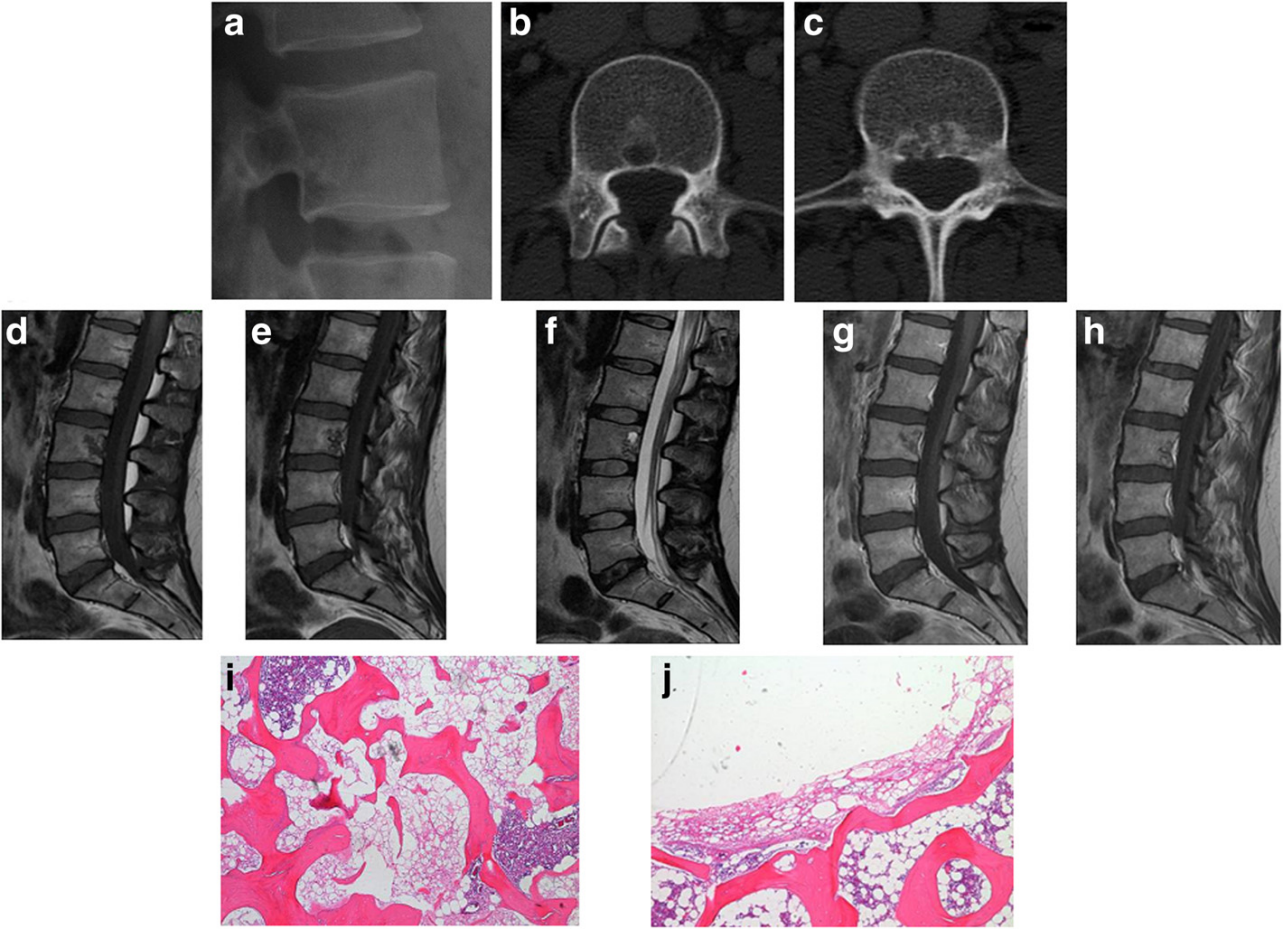
**Patient 1, a 33 year-old woman.** Images demonstrate radiodensity (sclerosis) (radiograph, **a**) as well as lytic areas (CT, **b** and **c**). The lesion is heterogeneous on both T1- **(d and e)** and T2-weighted **(f)** sequences and takes up contrast moderately **(g and h)**. The sclerotic aspect on imaging corresponds to classic areas of BNCT: permeative sheets of adipocyte-like tumor cells associated with bone sclerosis and mixed with normal bone marrow islands **(i)**. The round lytic area on imaging **(c)** corresponds to a centrally emptied notochordal cell lesion without evidence of chordoma at the periphery of the lesion **(j)**. The cystic nature of this lesion is best seen on T2-weighted imaging **(f)**.

**Figure 2:**
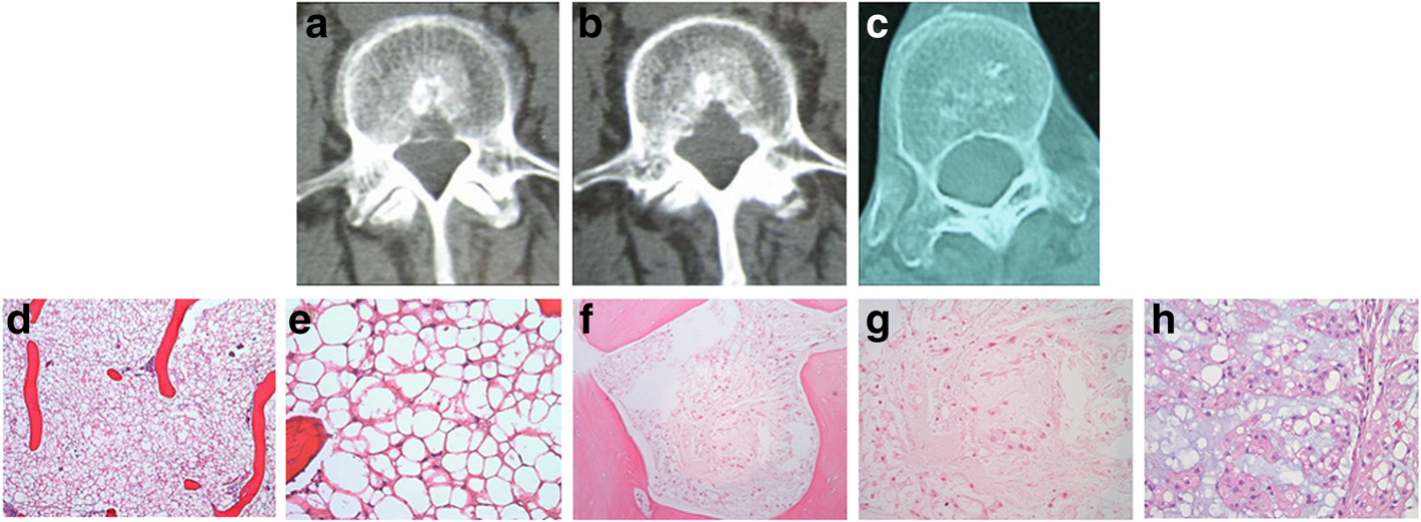
**Patient 2, a 57 year-old woman.** CT images reveal a mostly sclerotic lesion with focal areas of lysis, compromising the posterior cortex of L4 **(a and b)**. **c**: Similar lesion in the thoracic spine (discovered retrospectively, not biopsied); fat is visible inside the lesion. Microscopically, the BNCT component is composed of permeative sheets of cells (H&E, obj. 4, **d**). **e**: The cells are adipocyte-like without atypia (H&E, obj 20). **f**: A minute atypical area surrounded by osteosclerosis; higher magnification **(g)** reveals spindled notochordal cells with moderate atypia, but without a nodular growth pattern or clear myxoid stroma (H&E, obj 20). This lesion recurred as a classic chordoma **(h)**: cords and strands of tumor cells in a myxoid background with a nodular growth pattern (H&E, obj 10).

**Figure 3:**
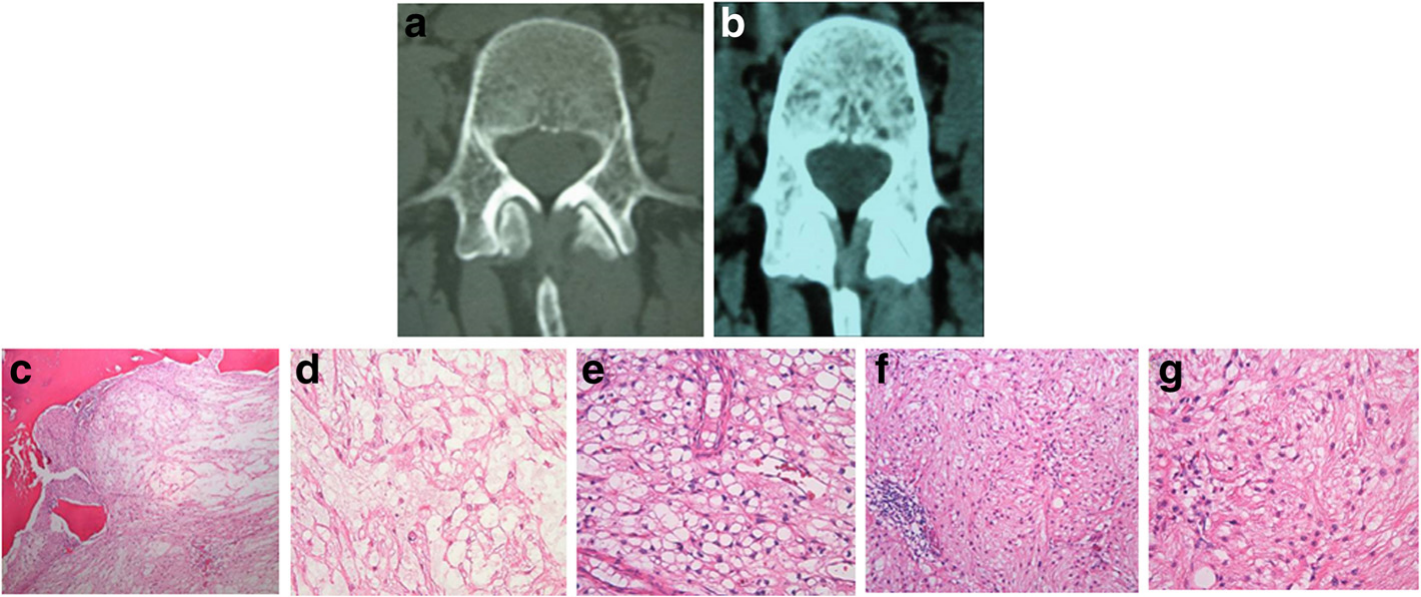
**Patient 3, 53 year-old woman. a** and **b**: CT demonstrates a mostly sclerotic lesion with small areas of lysis. **c**: The main part of the lesion is a chordoma, revealing the characteristic destructive nodular growth pattern of the tumor (H&E, obj 4). On higher power **(d)**, cords and strands of tumors cells in an abundant myxoid background with mild atypia are seen (H&E, obj 20). **e**: a small area of BNCT is composed of sheets of adipocyte-like cells, without a nodular growth pattern or myxoid background (H&E, obj 20). The atypical areas **(f and g)** do not fulfill the criteria for BNCT or chordoma. **f**: micronodular growth pattern, no myxoid background (H&E, obj 4). Eosinophilic spindle cells with mild atypia **(g)**.

**Figure 4:**
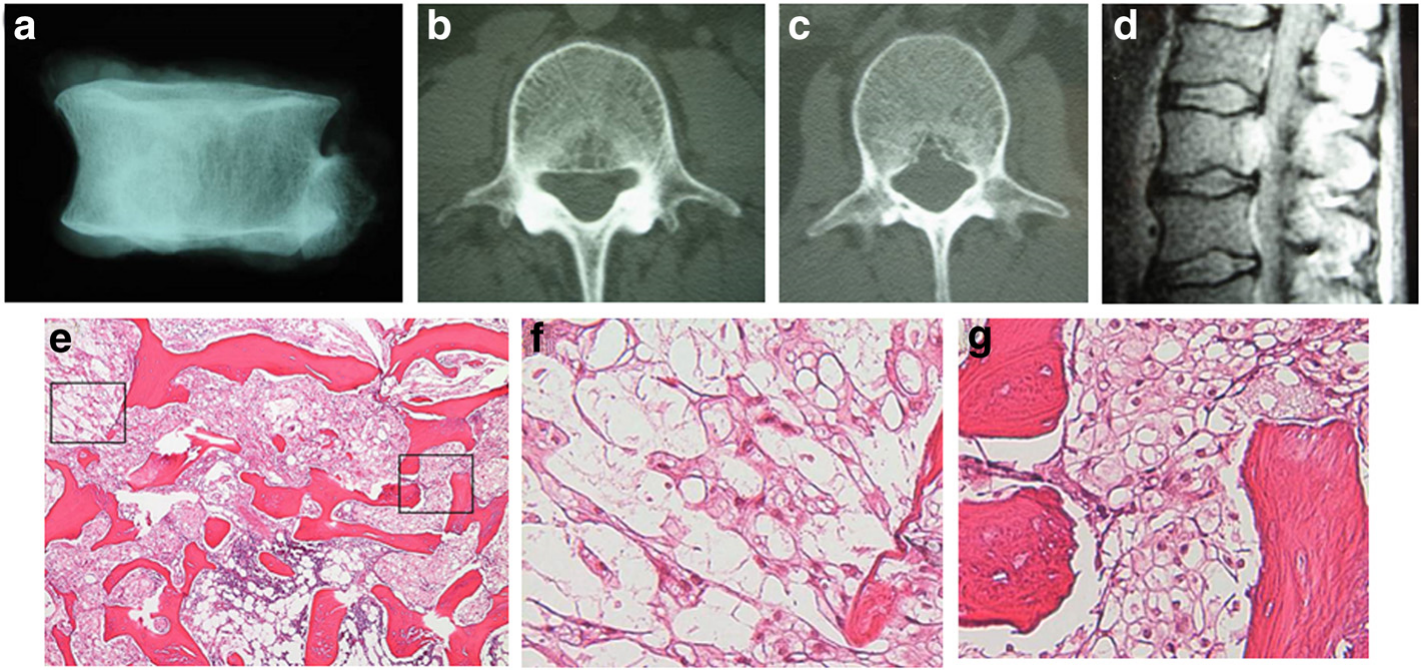
**Patient 4, 48 year-old male.** Radiograph of resected vertebra demonstrates a radiodense lesion **(a)**. On CT, areas of lysis are seen, portions of which compromise the posterior cortex **(b and c)**. This area corresponds to high signal on T2-weighted MRI **(d)**. **e**: Microscopically, atypical tissue transitions between chordoma (left) and BNCT (right) (H&E, obj 4). **f**: The chordomatous component is composed of cords of eosinophilic and vacuolated cells in a myxoid background (H&E, obj 10). **g**: The BNCT component consists of permeative sheets of adipocyte-like cells associated with bone sclerosis (H&E, obj 10).

There were three women, one man, aged 33–57 years (mean 48) at time of diagnosis. Re-evaluation yielded the following diagnoses: two BNCTs and two lesions with areas of both BNCT and chordoma (Figures 1, 2, 3 and 4).

All four patients had lesions located in the posterior aspect of lumbar vertebrae. All lesions were mild-moderately radiodense (sclerotic) on computed tomography (CT) with varying patterns and amounts of lysis. Two patients (1 and 4) had radiographs available for evaluation; the lesions were mildly radiodense. Of the patients who had magnetic resonance imaging (MRI) available for evaluation, one was heterogeneous on both T1- and T2-weighted sequences and enhanced with contrast administration (Patient 1). Patient 4 only had T2-weighted images available for evaluation; the lesion demonstrated high signal. All patients had radiologic signs of intralesional fat.

All four cases had areas that definitively met the pathologic criteria for BNCT. Patient 1 had classic features of BNCT as well as a well-limited cystic area, the inside border of which had layers of cells consistent with the rest of the lesion. This cystic portion was well-defined with a sclerotic border and without microscopic evidence of bone resorption. The lesion for Patient 2 was composed entirely of classic BNCT except for a minute atypical area. The other two cases were composed of distinct areas that definitively met the criteria for BNCT and for chordoma, as well as atypical areas that did not meet the criteria for either chordoma or BNCT. For all three lesions, the atypical areas seemed to "blend" or "flow" to/from the areas of BNCT and chordoma. None of the cases had microscopic evidence of extraosseous lesion extension.

## Discussion

Although intraosseous notochordal remnants have been recognized for over a century, interest was limited until recently. Focused studies as well as research in related topics such as degenerative disc disease have helped advance our collective knowledge of these lesions; [18-22] of utmost clinical importance is any relationship to chordoma. If all of these lesions naturally progress to chordoma or have the potential to transform into chordoma, they provide the opportunity to catch a malignancy at a very early, and small, stage.

Fundamental to any discussion of notochordal cell lesions is an understanding of notochord development. In humans, the notochord is a transient embryonic structure with critical structural and developmental roles [1,3,19]. After inducing vertebral column formation it disappears, leaving cellular remnants in the nucleus pulposus. There is later an apparent loss of notochordal cells such that they have rarely been reported beyond the first decade of life [2,3,18-22].

Notochordal remnants are occasionally found in fetal and adult vertebrae, with some suggesting this may be a more frequent occurrence than observations suggest [3,4,7,17,18,20,21]. It has been hypothesized that regression of the notochord may arrest at any point in development, leaving notochordal cells along its course that may then be subject to hyperplasia [1,3,8,17].

Extraosseous masses of benign vacuolated physaliferous cells at the base of the skull were identified as notochordal remnants in the mid-nineteenth century. Designated "ecchordosis physalifora", they were considered proliferation of ectopic tissue due to aberrant notochordal development and/or regression [1,3,6,8,17]. Malignant lesions with a similar morphology came to be called "chordoma"; believed to be of the same origin, it is a rare, slow-growing tumor with a poor prognosis, selectively affecting the axial skeleton [8,17,23].

Reports of intraosseous notochordal tissue were scant until the first modern histopathologic reports opened a broader discussion [1-6,8]. Initial questions focused on whether this tissue was a notochordal rest [4-6,8]. Based on size alone, these lesions had clearly grown beyond embryologic proportions; sclerotic trabeculae and the described permeative nature further implied growth. Some questioned whether a vestige could grow, stating that proliferation implies neoplasia or malignancy [5,6,8]. However, notochordal cells in the disc have been observed to have proliferative potential and ecchordosis physalifora, widely deemed benign, has been called a "proliferative rest" [3,8,17,21]. Moreover, it was argued that permeation is not a sine qua non for malignancy; that these lesions seemed to expand within the confines of the vertebrae without destruction while exhibiting permeative growth, some largely unchanged after years, supported the argument that they are benign [6,8]. Others argued that these may be characteristics of pre-chordomas or the early stages of very slow-growing chordomas [5,6,8]. In ensuing years, Japanese colleagues made significant contributions; they proffered that BNCTs were not merely the intraosseous counterparts to notochordal vestiges in discs but benign, slow-growing lesions which develop after birth, capable of malignant transformation [7,9-11].

Further questions regarding the origin and natural history of these lesions arose; did the notochordal cells migrate or herniate from the nucleus pulposus? Is it a lack of growth arrest as opposed to an initiation of growth? Is this true neoplasia or benign hyperplasia? As more cases came to light, various authors proposed radiologic and microscopic criteria to help distinguish BNCTs from chordoma [6-10,16].

### Radiology

Chordomas present as destructive, lytic lesions of bone on radiographs and CT, although intralesional calcification and varying radiodensity is reported [5,8,14,17,24]. Bony expansion may be seen. Bone scans demonstrate reduced or normal uptake [6,23]. MRI reveals low-intermediate signal on T1-weighted and high signal on T2-weighted sequences, usually with evidence of a large soft tissue mass [16,17,24]. Enhancement is common after contrast administration [14,16,17,24]. This tumor is slow-growing and progresses over time [6,8,17,23].

BNCTs of adequate size to be detected radiologically may or may not demonstrate mild to marked radiodensity (usually described as sclerosis) on radiographs and/or CT; the lesion is confined to the bone without expansion [6,8,10,16,17]. MRI is the most useful modality for detection. Most studies describe these lesions as low-intermediate signal intensity on T1-weighted images and intermediate-high signal intensity on T2-weighted images, occasionally heterogeneously so, with a lack of enhancement with contrast administration; [6,10,16,17,25] two authors report "poor" and definitive enhancement, respectively [6,11]. Bone scans are usually normal, however, cold spots at the location of the lesion have been reported [6,10,17]. Maintenance of trabecular architecture without bony destruction or expansion, lack of soft tissue extension, and a lack of enhancement after contrast administration have been reported as the most reliable means of distinguishing between BNCT and chordoma radiographically [8,10,16,17] (Table 1).

**Table 1:** Differences between BNCT and chordoma on imaging

	**BNCT**	**Chordoma**
Radiographs and CT	Sclerosis. Conservation of bone trabeculae	Lysis.
MR signal	Dark on T1, white on T2	Dark on T1, white on T2
MR after contrast medium injection	No change	Strong uptake
Cortex	Normal	Destroyed
Soft tissues	Normal	Invaded

### Pathology

That the embryonal notochord, notochordal remnants in the disc, ecchordosis physalifora, BNCT, and chordoma have remarkable histopathologic similarity is well-documented; diagnosis by light microscopy alone is difficult [1,3,6,8,22]. All contain univacuolated physaliferous cells and have similar immunohistochemical patterns, staining for S100, vimentin, EMA, and low-molecular weight cytokeratin and are negative for high-molecular weight cytokeratin [3,6-8,10,17]. Although cytokeratin 18 expression is now considered unreliable for differentiating between these lesions, brachyury, a transcription factor important in notochordal development, has been found to be a consistent marker for notochordal cell lesions [14,17,21,22,26].

Benign notochordal cell tumors are well-delineated lesions consisting of sheets or nests of physaliferous cells. These cells replace the bone marrow, filling the medullary spaces between bony trabeculae, but trabecular architecture is preserved. Hematopoietic islands or islands of marrow fat may be seen between the sheets of cells [6,9,10,17]. Trabeculae often demonstrate thickening, cement lines, and appositional or reactive new bone formation, correlating with the radiodensity often seen on CT and radiographs [5-9]. At low power, these cells may be confused with fat [6,10,14]. At high magnification, the characteristic physaliferous cells have a clear cytoplasm and eccentrically located oval or round nuclei. Also seen are less vacuolated cells with central, rounded nuclei and eosinophilic cytoplasm. Nuclear atypia is minimal; pleomorphism, mitoses, and necrosis are not seen [6,17]. Microcystic spaces with colloid-like material have been observed in some lesions [9,10]. Two of the key features differentiating these lesions are that they are not lobulated and lack a myxoid background [6]. Although soft tissue extension has not been reported radiologically, this lesion has been reported to extend extraosseously on microscopic examination [9,27].

Chordomas are characterized by a destructive growth pattern. Fibrous septae separate the lesion into lobules, within which are cords and strands of epithelial-like cells with clear, vacuolated cytoplasm (the physaliferous cells) or granular, eosinophilic cytoplasm, all set in a myxoid matrix. Atypia, pleomorphism, and mitotic figures may be mild or marked; necrosis may be seen and bony destruction is evident. Bone marrow and fat may be seen at the edge of the lesion but not within it. Chordomas exhibit a significant amount of histopathological variation from field to field, such that not all diagnostic features may be seen in a small specimen [6,8,17].

### Relationship between BNCT and chordoma

It has long been thought that chordomas might arise from notochordal remnants [1-4,8,17], with early arguments centered on their similar anatomical distributions [4,6,7,10,13,17,28]. As precursors, one would expect the incidence of rests to be higher than that of chordomas; chordomas occur at a rate of 0.08 per 100,000 while the incidence of BNCT has been reported as high as 20% [7,28], suggesting that if they are the precursors to chordoma, not all will transform.

Reports of BNCT and chordoma in the same person and even the same vertebra prompted several authors to propose that the chordomas had developed from the BNCT [1,11,13-16]. These authors described at minimum a sharp interface between, if not distinct separation of, the chordomatous and BNCT tissues, leading one group of authors to acknowledge that they could not rule out the possibility that the rare chordoma had arisen coincidentally with the more common BNCT [12].

Yamaguchi et al. detailed more compelling cases of microscopic foci of BNCT and chordoma in the same vertebrae of two elderly patients [12]. Referred to as "incipient chordomas" due to their size and because, although "occasionally arranged in a small lobular configuration", some classic aspects of chordoma were lacking, they concluded that the BNCT tissue had transformed. Certainly, these findings are very interesting. Both patients died of carcinoma; it is unclear if they had received chemotherapy, which another author suggested could cause morphological degeneration of notochordal remnants, possibly causing confusion [17]. Consistent with previous studies, the authors reported a sharp interface between the two tissue types.

### Unique features of our cases

Two of our cases were intraosseous chordomas without a soft tissue component (Patients 3, 4); these cases also had distinct areas of BNCT, as well as atypical tissue that failed to meet criteria for either BNCT or chordoma but instead seemed to be tissue in transition (Figures 3f, 4e). A third case was composed almost entirely of BNCT tissue with a very small atypical area (Figure 2f-g); this recurred as chordoma, despite an original vertebrectomy (Figure 2h). In each of these cases, there was "blending" or "flow" between the tissue types, without any sharp interfaces or clear demarcation to distinguish them (Figure 4e). Only one case failed to show any atypical areas or malignancy (Patient 1). It was distinctive for its macrocystic component, which had a well-defined sclerotic border, and for lack of any microscopic bone resorption, features consistent with a benign lesion (Figure 1).

These cases illustrate several important points. While the microscopic criteria for BNCT are well-described, the lines for the radiologic criteria seem to be ever more blurred. Several authors suggested that a key distinguishing feature of chordoma is the presence of a soft tissue mass; [10,16,17] on this basis, it seemed likely that all four of these intraosseous lesions initially diagnosed as chordoma would be BNCT. On the contrary, two of the four lesions had distinct chordomatous components. These findings demonstrate what makes sense intuitively: that very early stage chordomas will not demonstrate macroscopic - or any - soft tissue masses [5,8]. Likewise, a lack of contrast enhancement has been suggested as one of the primary means of differentiation between the two lesions [16]. However, chordomas and BNCTs have been reported to exhibit varying degrees of enhancement [6,11,24] and our case of BNCT demonstrated moderate enhancement (Figure 1g-h).

It has generally been accepted that lysis or evidence of bony destruction on imaging is inconsistent with a diagnosis of BNCT [8,10,16]. An interesting recent report of a BNCT demonstrated "bubbly" low-density areas with intact trabeculae surrounded by sclerosis on CT that corresponded to foci of increased signal on T1-weighted images [29]. These created a heterogeneous appearance on MRI and, after re-review of the authors' previous case with macrosection, were concluded to represent marrow inclusions. Although some authors previously reported heterogeneous MRI signal patterns for BNCT [14,25] and others appear to have similar findings [6,8,10], this was the first study to correlate imaging with a well-known microscopic characteristic of BNCT.

Similar findings were revealed in our cases; three had lesions with a comparable appearance: lucent areas with intact trabeculae, surrounded by sclerosis on CT (Figures 1c, 2a-c, 3a-b, 4b-c). Patient 1 also demonstrated the aforementioned heterogeneity and pattern on MRI (Figure 1d-f). However, the lytic macrocystic area seen on CT (Figure 1b) was only clearly evident on MRI on T2-weighted sequences (Figure 1f), and therefore did not represent marrow. It was difficult to appreciate on T1-weighted imaging (Figure 1d-e) and did not enhance with contrast (Figure 1g-h), indicating it might have been thick fluid. Certainly, it could be argued that perhaps the center of this lesion contained necrosis or myxoid material, possibly representing the beginnings of chordoma. As the cystic area was centrally empty on slides, it is impossible to be sure, however, the cells lining the cavity were BNCT without atypical areas or chordoma. In addition, its sclerotic border (Figure 1b, i) and the overall appearance of the lesion were consistent with the benign pathologic findings (Figure 1i-j).

More unique features were demonstrated in two of our cases with larger areas of lysis with compromise of trabecular architecture and destruction of the posterior cortex (Figures 2a-b, 4b-c). While this is not entirely surprising considering the microscopic evidence for chordoma in Patient 4, the lysis is fairly pronounced for Patient 2 considering mostly BNCT with only a minute focus of atypical tissue was found.

Portions of all four cases had the appearance of intralesional fat on CT (Figures 1c, 2a-c, 3a-b, 4b-c) and MRI (Figure 1e-f), a finding usually highly suggestive of a benign lesion [30]. Yet, two of these four cases had evidence of chordoma; one had lysis adjacent to the more benign-appearing area (Figure 4b-c), reflective of the pathology. Patient 3, however, had radiologic findings comparable with the aforementioned study [29], despite the main part of the lesion being chordoma.

Importantly, these cases underscore the significant difficulty in differentiating between BNCT and chordoma and the very real risk of misdiagnosis not only radiologically, but also histopathologically. Microscopic diagnosis is dependent on the proper specimen, made all the more challenging by the usual sampling technique and size for these lesions (needle biopsy through the pedicle) and by the microscopic heterogeneity of chordoma [6,8,17]. Patient 2 illustrates just how easily the diagnosis of chordoma can be missed; the atypical area was insufficient to meet the criteria for chordoma (Figure 2f-g). Moreover, this area was an extremely small portion of the overall sample, easily able to be missed. Was this a sampling error of a lesion that was already a chordoma or was this the most "malignant" portion of the lesion on gross examination, appropriately sampled and evaluated, and it was only over time that it transformed into chordoma?

These atypical areas may be sites of active malignant transformation or precursor, transitional tissue. It is possible that there are variants of BNCT, some already early chordomas or predetermined to become chordoma, others destined to remain benign. Or perhaps, given the rarity of chordomas relative to BNCTs, all BNCTs have the potential to transform into chordoma under the right circumstances, such as an environmental insult or chromosomal alteration; recent studies have implicated gene amplification and duplication in chordoma development, with attention focused on Sonic hedgehog and T (brachyury) genes [15,20,21,23,26]. That chordomas are rare before mid-life but benign notochordal tissue has been found in vertebrae at all ages further supports this hypothesis [3,4,8-10,23,25,28].

Morphology is confusing; fetal notochordal tissue has cords and nests in a myxoid background like chordoma, however, BNCT lacks these features. These differences may be explained by different local environments such as bone marrow, which may promote proliferation, possibly because of pressure or vascularity differences, growth factors, or some combination thereof or, perhaps, as some authors contend, these lesions arise de novo as notochordal-type tissue expressing markers like brachyury [10,21,22,25]. Similarly, it has been suggested that the widespread belief that chordomas are of notochordal origin may be incorrect [17], that they may originate from another cell type, expressing genes and proteins consistent with notochordal cells. However, a recent fate-mapping study in mice demonstrated notochordal remnants in the vertebrae or, rarely, the annulus fibrosis; they were found along the entire vertebral column, were seen in all animals studied, and remained throughout life [20]. If a similar process takes place in humans and these remnants remain dormant for years, then the transitional atypical tissue seen in our study is likely benign to malignant transformation in progress.

It is our opinion that BNCTs are notochordal remnants that have the potential for malignant transformation. They may grow with the vertebrae, occasionally becoming quite large, but retaining their benign characteristics. That several cases have not changed substantially over years, coupled with the high incidence of BNCTs relative to chordomas, Horwitz' comprehensive work, and the aforementioned lineage study underscore their benign nature [3,6,8,10,17,20].

Investigations over the last three decades have shed significant light on these intraosseous foci of notochordal tissue. While there remains much to be elucidated, our cases strengthen the argument for a relationship between BNCT and chordoma; the BNCT that recurred as a chordoma is certainly provoking, but not yet sufficient to state it was a purely benign-to-malignant transformation. It is encouraging to think that chordoma may be caught in its earliest stages, thus reducing morbidity for the patient. If BNCT is found more frequently than one in five patients, as some suspect [7,13,20], this poses an important question for clinicians: are all BNCT to be followed routinely? If yes, over what intervals and for how long? At present no one radiologic aspect confirms a diagnosis. It is change of the lesion over time that appears to be the most helpful radiologic sign, with the development of bony destruction, lysis, enhancement with contrast, and/or a soft tissue mass to be the most worrisome indication of chordoma. Above all, close correlation between radiologic and histopathologic findings is critical.

## Conclusion

We present four cases that illustrate ongoing challenges in the diagnostic differentiation between BNCT and chordoma. All of the cases had unique features on imaging and three had atypical areas on histopathology. While we maintain that BNCT is itself a benign entity, the microscopic blending of these atypical areas with BNCT and/or chordoma strengthens the argument for a relationship between the two entities and supports the idea that some BNCT may progress to chordoma. Our study highlights the need for adequate imaging and sampling and close correlation between radiology and histopathology. It dispels the notion that any single radiologic criterion to distinguish between BNCT and chordoma is reliable and opens the discussion as to whether or how to monitor BNCTs.

## Competing interest

The authors declare that they have no competing interest.

## Authors’ contribution

JK wrote the article. FL checked the histology and the text. PP Checked the list of all patients and the quality of the work. SB was the surgeon who operated the patients, and checked the article. JM proposed the idea of the article and checked the histology. BM and EB checked the work. DV coordinated and checked the article. All authors read and approved the final manuscript.
